# Production of Lentiviral Vectors Using a HEK-293 Producer Cell Line and Advanced Perfusion Processing

**DOI:** 10.3389/fbioe.2022.887716

**Published:** 2022-06-14

**Authors:** Michelle Yen Tran, Amine A. Kamen

**Affiliations:** Viral Vectors and Vaccines Bioprocessing Group, Bioengineering Department, McGill University, Montreal, QC, Canada

**Keywords:** lentiviral vector, stable producer cell line, lentiviral vector production, perfusion, cell retention device, lentiviral vector manufacturing, large-scale manufacturing, continuous manufacturing

## Abstract

The field of lentiviral vector (LV) production continues to face challenges in large-scale manufacturing, specifically regarding producing enough vectors to meet the demand for treating patients as well as producing high and consistent quality of vectors for efficient dosing. Two areas of interest are the use of stable producer cell lines, which facilitates the scalability of LV production processes as well as making the process more reproducible and robust for clinical applications, and the search of a cell retention device scalable to industrial-size bioreactors. This manuscript investigates a stable producer cell line for producing LVs with GFP as the transgene at shake flask scale and demonstrates LV production at 3L bioreactor scale using the Tangential Flow Depth Filtration (TFDF) as a cell retention device in perfusion mode. Cumulative functional yields of 3.3 x 10^11^ and 3.9 x 10^11^ transducing units were achieved; the former over 6 days of LV production with 16.3 L of perfused media and the latter over 4 days with 16 L. In comparing to a previously published value that was achieved using the same stable producer cell line and the acoustic filter as the perfusion device at the same bioreactor scale, the TFDF perfusion run produced 1.5-fold higher cumulative functional yield. Given its scale-up potential, the TFDF is an excellent candidate to be further evaluated to determine optimized conditions that can ultimately support continuous manufacturing of LVs at large scale.

## 1 Introduction

Lentiviral vectors (LVs) are a popular gene delivery tool in cell and gene therapy and they are the primary tool for *ex vivo* transduction of T-cells for expression of chimeric antigen receptor in CAR-T cell therapies (Escors et al., 2012; Naldini et al., 2016; Milone and O’Doherty, 2018). In LV production, there have been many improvements in vector design, upstream processing, and downstream processing over the years (Tolmachov et al., 2011; Segura et al., 2013; McCarron et al., 2016; Merten et al., 2016; Sharon and Kamen, 2018; Moreira et al., 2021; Perry and Rayat, 2021). However, the field continues to face difficulties in large-scale manufacturing of LVs (Ansorge et al., 2009; Ansorge et al., 2010; Martínez-Molina et al., 2020), specifically regarding producing enough vectors to meet the demand for treating patients as well as producing high and consistent quality of vectors for efficient dosing. Continuous processing is appealing for viral vectors, enzymes, exosomes, and cell-based therapies that are unstable (McCarron et al., 2016; Schofield, 2018). For example, the stability of LVs is relatively low, with a half-life of only 3–18 h at 37°C (Ansorge et al., 2010), and LVs have a high sensitivity to environmental pH, salt concentration, and shear stress during harvest and downstream processing (Merten et al., 2016). In addition, LVs lose their functionality significantly in accordance with downstream processing steps (Transfiguracion et al., 2020) and process hold times.

An impactful strategy for process intensification is to establish an integrated continuous processing, where continuous harvesting in perfusion mode is combined with downstream capture, to provide benefits such as improved product quality and faster processing time, which reduces cost and increases flexibility and productivity (Gutiérrez-Granados et al., 2018; Moleirinho et al., 2020). In terms of LV production, these improvements would have a direct impact on LV product quality in two ways–first, increasing vector production by lengthening the LV production phase through the means of perfusion; second, and more importantly, increasing vector quality by slowing down the loss of functionality of LVs through reduced processing and hold times, which is achieved by continuously removing LVs from the cell culture environment and passing the LVs directly on to downstream processing steps.

To date, no such strategy has been successfully implemented for LV production, largely because commercial cell retention devices used for perfusion tend to retain the lentivirus product, rendering it difficult to remove the fragile LVs. Although a proof of principle has been demonstrated using an acoustic filter (Manceur et al., 2017), this system is not scalable to industrial-size bioreactors. This manuscript explores the possibility of utilizing the Tangential Flow Depth Filtration (TFDF) device as a cell retention device to support LV production in perfusion mode at manufacturing scale, since the device is scalable up to 2000L bioreactor scale. The TFDF combines the benefits of both tangential flow and depth filtration, where it can process high cell density cultures with minimal membrane fouling while allowing for high product recovery. The TFDF is currently commercially used for clarification to replace centrifugation or reduce depth filters and it has been shown to effectively separate cells and cell debris from LVs (Williams et al., 2020). In addition, this device has shown to be non-stressful on cells while supporting multiple harvests (Williams et al., 2020), which supports the notion that it can be used in perfusion mode during cell culture.

Traditionally, LVs are produced by transient transfection, using 3 to 4 plasmids. Due to manufacturing scalability challenges, packaging cell lines have been developed by stably integrating necessary genetic elements for the assembly and functioning of the vectors, leaving only the transgene plasmid to transfect (Kafri et al., 1999; Hu et al., 2015). To further facilitate the scalability of LV production processes, producer cell lines have been developed to integrate the remaining transgene plasmid, making the process more reproducible for clinical applications (Sanber et al., 2015; Tomás et al., 2018; Chen et al., 2020). The stable producer cell line HEK293SF-LVP-CMVGFPq-92 (Manceur et al., 2017), abbreviated as Clone 92, is utilized for the work presented in this manuscript. These producer cells contain GFP as the transgene and VSV-G at the membrane surface; and they are induced with doxycycline and cumate only during the time of LV production, which addresses cytotoxicity issues that typically arise from the viral proteins (e.g., Gag, Rev, VSV-G) (Broussau et al., 2008).

This manuscript presents LV production (1) under different culturing parameters at shake flask scale in batch and pseudo-perfusion modes to better understand Clone 92 producer cells and (2) in perfusion mode at 3L bioreactor scale with TFDF as the cell retention device to evaluate its performance. Of the main aspects that contribute to the LV product quality–identity, potency, purity, and safety–the analysis for this work mainly focuses on the potency aspect, as the identity of the LVs produced by this producer cell line has already been characterized and published (Transfiguracion et al., 2020), and purity and safety are typically addressed in downstream processing. To probe the potency of the LVs produced in this manuscript, we assess the functional vector particles and total vector particles. We use the gene transfer assay, a cell-based analytical method that measures the transgene expression in transduced target cells, to report functional vector particles in transducing units. We use the droplet digital PCR, a physical-based analytical method, to report total vector particles in vector genome units.

## 2 Materials and Methods

### 2.1 Stable Producer Cell Line

The stable producer cell line HEK293SF-LVP-CMVGFPq-92 (abbreviated as Clone 92) (Manceur et al., 2017), developed by the National Research Council Canada, was used to produce LVs that contain GFP as the transgene and VSV-G at the membrane surface by inducing with 1 μg/ml final concentration of doxycycline and 10 μg/ml final concentration of cumate. The combination of the Tet-on system and the cumate switch provides tighter transcription regulation (Broussau et al., 2008). The Tet-on system is based on the addition of the tetracycline/doxycycline antibiotic in the culture medium to trigger gene transcription through the tetracycline response element by promoting the binding of the reverse tetracycline transactivator (rtTA2s-M2) to the tetracycline promoter (TR5). The addition of cumate releases the cumate repressor from the copper oxide promoter, allowing for transcription (Broussau et al., 2008).

### 2.2 LV Production at Shake Flask Scale

125 ml polycarbonate shake flasks from Avantor (Phillipsburg, NJ) were used to produce LVs at shake flask scale. Clone 92 cells were inoculated at 0.35 × 10^6^ cells/mL in Hyclone HyCell TransFx-H media from Cytiva (Marborough, MA), supplemented with final concentrations of 4 mM GlutaMax from life Technologies (Grand Island, NY) and 0.1% Kolliphor poloxamer 188 from Millipore Sigma (Ontario, Canada), with a working volume of 25 ml. The shake flasks were incubated at 37°C with shaking speed of 135 RPM. After 48 h, the low cell density flasks reached 1 × 10^6^ cells/mL and they were induced with 1 μg/ml final concentration of doxycycline hyclate and 10 μg/ml final concentration of 4-isopropylbenzoic acid, both from Sigma-Aldrich (Darmstadt, Germany). The high cell density flasks either went through a one-step concentration using centrifugation or daily medium exchange to reach higher cell density, before being induced for LV production.

Some flasks were subjected to medium exchange after induction to achieve “pseudo-perfusion” and some flasks had basic feeding strategies of 6 g/L glucose, prepared in-house using D-(+)-Glucose from Sigma-Aldrich (Darmstadt, Germany), and 3 mM GlutaMax from life Technologies (Grand Island, NY) from the time of induction until the time of harvest. For the flasks that underwent medium exchange after induction, the inducers doxycycline hyclate and 4-isopropylbenzoic acid were added in the medium at the values previously defined above to allow the continuation of LV production. The LVs were harvested 3 days post induction by centrifugation at 300 *g* for 5 min to remove the induced Clone 92 cells, then 1200 g for 10 min to collect the supernatant. Cell count was done using the Vi-Cell XR Cell Viability Analyzer from Beckman Coulter (Indianapolis, IN).

The details of the different tested parameters are well described in the results Section 3.1 as well as Table 1. The first set of shake flask experiments (M1 through M6) for parameter evaluation were run in single flasks and the second set of shake flask experiments (M17 through M25) for parameter confirmation were run in triplicate flasks.

**TABLE 1:** Comparison of LV production at shake flask scale.

Shake flask	Parameters	TFP = total functional particles (in transducing units, TU)				TVP = total vector particles (in vector genome copies, Vg)				TVP/TFP
		1 dpi	2 dpi	3 dpi	Final product	1 dpi	2 dpi	3 dpi	Final product	Final product
A1. Parameter evaluation: Comparing to M1 (baseline conditions: LCD, batch, no feed)										M1: 55
M2	LCD	+3.4x	+15x	+3.9x	+6.1x	+5.2x	+2.5x	+5.4x	+3.1x	27 (-2.0x)
	Pseudo-perfusion									

M3	HCD-C	+25x	+41x	+8.4x	+15x	+7.8x	+6.6x	+3.0x	+5.1x	19 (-2.9x)

	Feed									
M4	HCD-ME	+41x	+35x	+4.7x	+11x	+40x	+9.0x	+0.4x	+5.8x	28 (-2.0x)

	Feed									
M5	HCD-ME	+37x	+52x	+8.2x	+17x	+31x	+9.7x	+7.6x	+4.9x	16 (-3.4x)
	Pseudo-perfusion									

M6	HCD-ME	+35x	+111x	+13x	+32x	+25x	+17x	+9.6x	+13x	23 (-2.4x)
	Pseudo-perfusion									
	Feed									
A2. Parameter evaluation: Comparing one-step concentration vs. medium exchange to reach HCD										
M3	Concentration		comparable	+1.8x	+1.3x			+7.2x	comparable	-1.5x
M4	Medium exchange	+1.6x				+5.1x	+1.4x			
A3. Parameter evaluation: Effect of pseudo-perfusion at HCD										
M4		comparable				+1.6x				
M6	Pseudo-perfusion		+3.1x	+2.8x	+2.8x		+1.9x	+23x	+2.3x	-1.2x
A4. Parameter evaluation: Effect of feed at HCD										
M5		comparable				+1.2x				-1.4x
M6	Feed		+2.2x	+1.6x	+1.9x		+1.7x	+1.3x	+2.7x	
B. Parameter confirmation: Comparing the average of two selected parameter sets vs. baseline conditions set (i.e., LCD set)										LCD: 60
HCD-C set (*n* = 3)	HCD-C	+13x	+40x	+15x	+23x	+10x	+12x	+3.6x	+8.6x	22.3 (-2.7x)
	Pseudo-perfusion, Feed									
HCD-ME set (*n* = 3)	HCD-ME	+71x	+46x	+9.2x	+26x	+52x	+15x	+6.0x	+12x	29.8 (-2.0x)
	Pseudo-perfusion, Feed									

TFP, total functional particles; TVP, total vector particles; TU, transducing units; Vg, vector genome; dpi, days post induction; LCD, low cell density; HCD-C, high cell density, obtained by one-step concentration; HCD-ME, high cell density, obtained by daily medium exchange; pseudo-perfusion = daily medium exchange after induction to mimic perfusion at bioreactor scale; feed = 6 g/L glucose and 3 mM glutamine daily. Parameter evaluation was implemented in single flasks (M1 through M6) to explore different parameters (inducing at HCD, pseudo-perfusion, and feeding) to select the best ones leading to improved yields for LV production using Clone 92 producer cells. Parameter confirmation was implemented in triplicate flasks for 3 sets (LCD baseline conditions; HCD-C + pseudo-perfusion + feed; HCD-ME + pseudo-perfusion + feed) to confirm results.

### 2.3 LV Production at Bioreactor Scale—Batch Mode

Two 1L bioreactors from Applikon Biotechnology (Delft, Netherlands) were used to produce LVs in batch mode. Clone 92 cells were inoculated at 0.35 × 10^6^ cells/mL in Hyclone HyCell TransFx-H media from Cytiva (Marborough, MA), supplemented with final concentrations of 4 mM GlutaMax from life Technologies (Grand Island, NY) and 0.1% Kolliphor poloxamer 188 from Millipore Sigma (Ontario, Canada), at a working volume of 700 ml. Some media was added 48 h after inoculation to target 1 × 10^6^ cells/mL at the time of induction, 1 μg/ml final concentration of doxycycline hyclate and 10 μg/ml final concentration of 4-isopropylbenzoic acid, both from Sigma-Aldrich (Darmstadt, Germany), were added for induction, and the final working volume was at 850 ml. The LVs were harvested 3 days post induction by centrifugation at 300 *g* for 5 min to remove the induced Clone 92 cells, then 1200 g for 10 min to collect the supernatant. The set points were 7.15 for pH, 37°C for temperature, 40% for dissolved oxygen, and 100 rpm for stirrer. Cell count was done using the Vi-Cell XR Cell Viability Analyzer from Beckman Coulter (Indianapolis, IN).

### 2.4 LV Production at Bioreactor Scale—Perfusion Mode

Two 3L bioreactors from Applikon Biotechnology (Delft, Netherlands) were used for LV production in perfusion mode with the Tangential Flow Depth Filtration (TFDF) cartridge from Repligen Corp (Rancho Dominguez, CA) as the cell retention device, which was operated by the KML-100 System, also from Repligen Corp. Clone 92 cells were inoculated at 0.35 × 10^6^ cells/mL at 2 L working volume and the cells were grown in batch mode until 72 h after inoculation. Then, perfusion started at 0.5 VVD (vessel volume per day) for 3 days and ramped up to 0.75 and 1 VVD, respectively, for the following 2 days to support the high cell density. At 176 h after inoculation, in which the viable cell density was 11.4 × 10^6^ cells/mL for perfusion run 1 (P1) and 12.3 × 10^6^ cells/mL for perfusion run 2 (P2), 1 μg/ml final concentration of doxycycline hyclate and 10 μg/ml final concentration of 4-isopropylbenzoic acid, both from Sigma-Aldrich (Darmstadt, Germany), were added for induction and perfusion continued at 1 VVD for P1 and ramped up to 2 VVD for P2.

The inducers and basic feeding strategies of 6 g/L glucose, prepared in-house using D-(+)-Glucose from Sigma-Aldrich (Darmstadt, Germany), and 3 mM GlutaMax from life Technologies (Grand Island, NY) were employed from the time of induction until the final harvest, which was at 6 days post induction at 3.3 × 10^6^ cells/mL for P1 and 4 days post induction at 5.9 × 10^6^ cells/mL for P2. The HyClone HyCell TransFx-H modified SH31192 (referred to as “Prototype” media in the manuscript) from Cytiva (Logan, UT) was used for the cell growth phase in both perfusion runs (VVD: 0.5, 0.5, 0.75, 1). For P1, the HyCell TransFx-H medium from Cytiva (Marborough, MA) was used for the LV production phase (VVD: 1, 1, 1.25, 1.25, 1.25, 1). For P2, the HyClone HyCell TransFx-H modified SH31192 was used for the LV production phase at a constant 2 VVD rate each day. Both types of HyCell media were supplemented with final concentrations of 4 mM GlutaMax from life Technologies (Grand Island, NY) and 0.1% Kolliphor poloxamer 188 from Millipore Sigma (Ontario, Canada).

For P1, one TFDF cartridge with the surface area of 30 cm^2^ was used for cell growth phase (7 days and 8 h), LV production phase (6 days), and a final harvest step (1.5 h), where the cell culture in the bioreactor was concentrated, diafiltered with 1x phosphate buffered saline (PBS) from Cytiva (Logan, UT), and concentrated again. For P2, one TFDF cartridge with the surface area of 30 cm^2^ was used for cell growth phase (7 days 8 h) and LV production phase (4 days), with no final harvest step. The set points for the bioreactors were 7.15 for pH, 37°C for temperature, 40% for dissolved oxygen, and 100 rpm for stirrer. Cell count was done using the Vi-Cell XR Cell Viability Analyzer from Beckman Coulter (Indianapolis, IN).

### 2.5 Gene Transfer Assay for Functional LV Quantification

A flow cytometry based GTA was used to determine functional vector titer in transducing units per milliliter (TU/mL). HEK293SF (Cote et al., 1998) cells were inoculated in tissue culture 24-well suspension plates from Sarstedt (Nümbrecht, Germany) at 0.5 × 10^6^ cells/mL in Hyclone HyCell TransFx-H media from Cytiva (Marborough, MA) supplemented with 8 ng/μL final concentration of polybrene at a volume of 450 µL per well and then transduced with 50 µL neat or diluted LVs per well, for a final volume of 500 µL per well. LV sample dilutions were made with HyCell TransFx-H media. The plates were incubated for 72 h at 37°C with shaking speed of 135 RPM.

Transduced cells were harvested by centrifugation at 500 *g* for 6 min to remove the supernatant. The pellet of transduced cells was resuspended in 150 µL of 2% paraformaldehyde (PFA) from Electron Microscopy Sciences (Hatfield, PA) in 1x phosphate buffered saline (PBS) from Cytiva (Logan, UT) for fixing cells for 30 min. The transduced cells were once again centrifuged at 500 g for 6 min and resuspended in 150 µL of 1x PBS before reading for GFP expression. Flow cytometry was carried out on the Accuri C6 instrument from BD Sciences (Franklin Lakes, NJ).

All LV samples, positive control, and negative control were run in duplicates on the same assay plate. The LV samples were assayed at the same dilution in duplicates. The positive control was an LV supernatant produced in our lab that has been used as the internal control for our GTA and ddPCR work. The negative control was cells that were “transduced” with 50 µL media rather than LVs. The percent of GFP expression was used to calculate for the titer in TU/mL. The equation used for the calculation is (GFP percentage)/100 x total cells x dilution factor x 1000/(volume of LVs).

### 2.6 Droplet Digital Polymerase Chain Reaction Assay for Total LV Quantification

The LV vector genome (Vg/mL) was quantified by a QX200™ Droplet Digital PCR (ddPCR) System from Bio-Rad (Hercules, CA). Prior to running samples on the ddPCR system, sample preparation included extracting RNA from LV samples using the High Pure Viral Nucleic Acid Kit from Roche (Basel, Switzerland) and reverse transcribing into cDNA using the iScriptTM Select cDNA Synthesis Kit from Bio-Rad (Hercules, CA), both following the manufacturers’ protocols. Although the Select cDNA Synthesis Kit states that 1pg to 1 µg of total RNA can be accommodated in the cDNA prep, results from our lab showed that either extreme of that range gives falsely elevated vector genome titer. Thus, to minimize variability, we included a normalization step by targeting 10 ng RNA for cDNA synthesis for every sample.

Following the RNA extraction step, the NanoDrop™ 2000 Spectrophotometer from Thermo Fisher Scientific (Waltham, MA) was used to determine the RNA content and the elution buffer from the High Pure Viral Nucleic Acid Kit was used as a buffer blank. The RNA samples were read in duplicates on the NanoDrop to ensure reliable values. Then, the RNA samples were diluted 1:100 with milli-Q water, and 10 ng RNA from that dilution was used in the cDNA synthesis step for each sample. For example, for a sample where a value of 100 ng was obtained from the NanoDrop and 1 ng was obtained from the 1:100 dilution, 10 µL of the 1:100 dilution plus 3 µL milli-Q water was used for a total sample volume of 13 µL in the cDNA reaction.

Each PCR reaction was prepared with 11.1 µL of the QX200™ ddPCR™ EvaGreen Supermix, 1.1 µL of the 2 µM stock of woodchuck hepatitis virus posttranscriptional regulatory element (WPRE) primer set, 4.4 µL milli-Q water, and 5.5 µL neat or diluted cDNA samples in milli-Q water. WPRE was used because it is known to stabilize the transgene mRNA and therefore enhance transgene expression delivered by LVs (Zufferey et al., 1999). For droplet generation in the QX200™ Droplet Generator, 20 µL of the PCR reactions were transferred to the G8™ Cartridges for QX200™. Then, the droplets were transferred to the ddPCR™ 96-Well Plate and the following PCR program was run on the thermo-cycler: one cycle of 95°C for 5 min; 40 cycles of 95°C for 30 s, 60°C for 1 min, and 72°C for 30 s; one cycle of 72°C for 5 min; indefinite 12°C hold. ddPCR results were analyzed with the QX200™ Droplet Reader and QuantaSoft Program.

All LV samples, positive control, and negative control were run in duplicates. LV samples were run in duplicates at two different sample dilutions. The positive control was an LV supernatant produced in our lab that has been used as the internal control for our ddPCR and GTA work. The negative control was milli-Q water that is added in place of an LV sample. Reverse transcription minus controls were tested to ensure there is no detectable genomic DNA impurity from the producer cells present in the LV production batches.

### 2.7 Picogreen Assay for DNA Quantification

DNA was quantified using the Quant-iT PicoGreen dsDNA Assay kit from Invitrogen (Eugene, OR). Each LV sample was serially diluted from 1:2 to 1:256 with 1x TE buffer in the Corning™ Polystyrene 96-Well Microplate from Fisher Scientific (Ontario, Canada). The λ dsDNA standard was diluted with 1x TE buffer from 0 to 500 ng/ml and the dilutions were included in duplicates on the same plate. After adding the diluted dye reagent to each well and incubating for 15 min at room temperature, the fluorescence was measured in the SYNERGY HTX multi-mode reader at 480/520 nm. The final DNA concentrations for samples were calculated based on the generated standard curve.

### 2.8 Statistical Analysis

Ordinary one-way ANOVA was performed to compare the effect of the selected parameters (i.e., high cell density at the time of induction, medium exchange post-induction to implement pseudo-perfusion, and feeding post-induction) on the total functional particles and total vector particles for the second set of shake flask experiments as described in Section 3.1. An unpaired *t*-test was performed to compare the total functional particles and total vector particles attained in perfusion mode and batch mode at bioreactor scale as described in Section 3.3. For all analysis, the alpha was set to 95%, the comparisons that resulted in *p* values <0.05 were considered statistically significant, and the representation of the *p*-values in the figures is as follows: 0.1234 (ns), 0.0332 (*), 0.0021 (**), 0.0002 (***), <0.0001 (****).

## 3 Results

### 3.1 LV Production at Shake Flask Scale

An extensive set of shake flask experiments was performed to explore different parameters to select the best ones leading to improved yields for LV production using Clone 92 producer cells. The tested parameters are low cell density (LCD) of 1 × 10^6^ cells/mL versus high cell density (HCD) of 5 × 10^6^ cells/mL at the time of induction (TOI), one-step concentration versus medium exchange before induction as the method in reaching HCD, medium exchange after induction to mimic perfusion at bioreactor scale (henceforth referred to as “pseudo-perfusion,” as the medium is exchanged every 24 h instead of continuously), and basic feeding strategies of 6 g/L glucose and 3 mM glutamine after induction.

Comparing to the baseline parameters of LCD at the TOI with no medium exchange (i.e., batch mode) and no feeding (baseline shake flask referred to as M1), the first set of experiments was designed to assess: whether medium exchange post-induction (i.e., pseudo-perfusion) improve LV production at the same LCD (M2), whether Clone 92 cells can produce LVs at HCD with only feeding if a one-step concentration method is employed (M3), whether there are differences in one-step concentration (M3) versus medium exchange (M4) pre-induction as a means of reaching HCD, and the effects of pseudo-perfusion and feeding (M5 and M6).

Table 1 compares the total functional particles (TFP) in transducing units (TU), determined by the gene transfer assay (GTA) with GFP as the readout signal, and the total vector particles (TVP) in vector genome copies (Vg), determined by droplet digital PCR, for the sample pools at 1 dpi (day post induction), 2 dpi, and 3 dpi, as well as final product pools. Table 1 also compares the ratio of TVP to TFP (TVP/TFP) for the final product pools, where a smaller number indicates higher LV potency since it means that a higher percentage of the produced total vector particles have effectively transduced the host cells and delivered the genetic material to be integrated in the host cell genome, thus expressing the GFP transgene.

In Table 1, Section A1 summarizes the increase or decrease of TFP, TVP, and TVP/TFP of shake flasks M2-M6 with the tested parameters, as described in the preceeding paragraph, when compared to the basline shake flask M1. These results show that the strategies of inducing at HCD, pseudo-perfusion, and feeding can increase the TFP and TVP of the final product up to 32- and 13-fold, respectively, and decrease the TVP/TFP up to 3.4-fold. Section A2 shows that one-step concentration increases the TFP of the final product by 1.3-fold and decreases the TVP/TFP by 1.5-fold when compared to medium exchange pre-induction to reach HCD, while the TVP is comparable. Section A3 shows that pseudo-perfusion alone at HCD increases the TFP and TVP of the final product by 2.8- and 2.3-fold, respectively, and decreases the TVP/TFP by 1.2-fold. Section A4 shows that feeding alone at HCD increases the TFP and TVP of the final product by 1.9- and 2.7-fold, respectively, although the TVP/TFP is in favor of no feeding.

**FIGURE 1:**
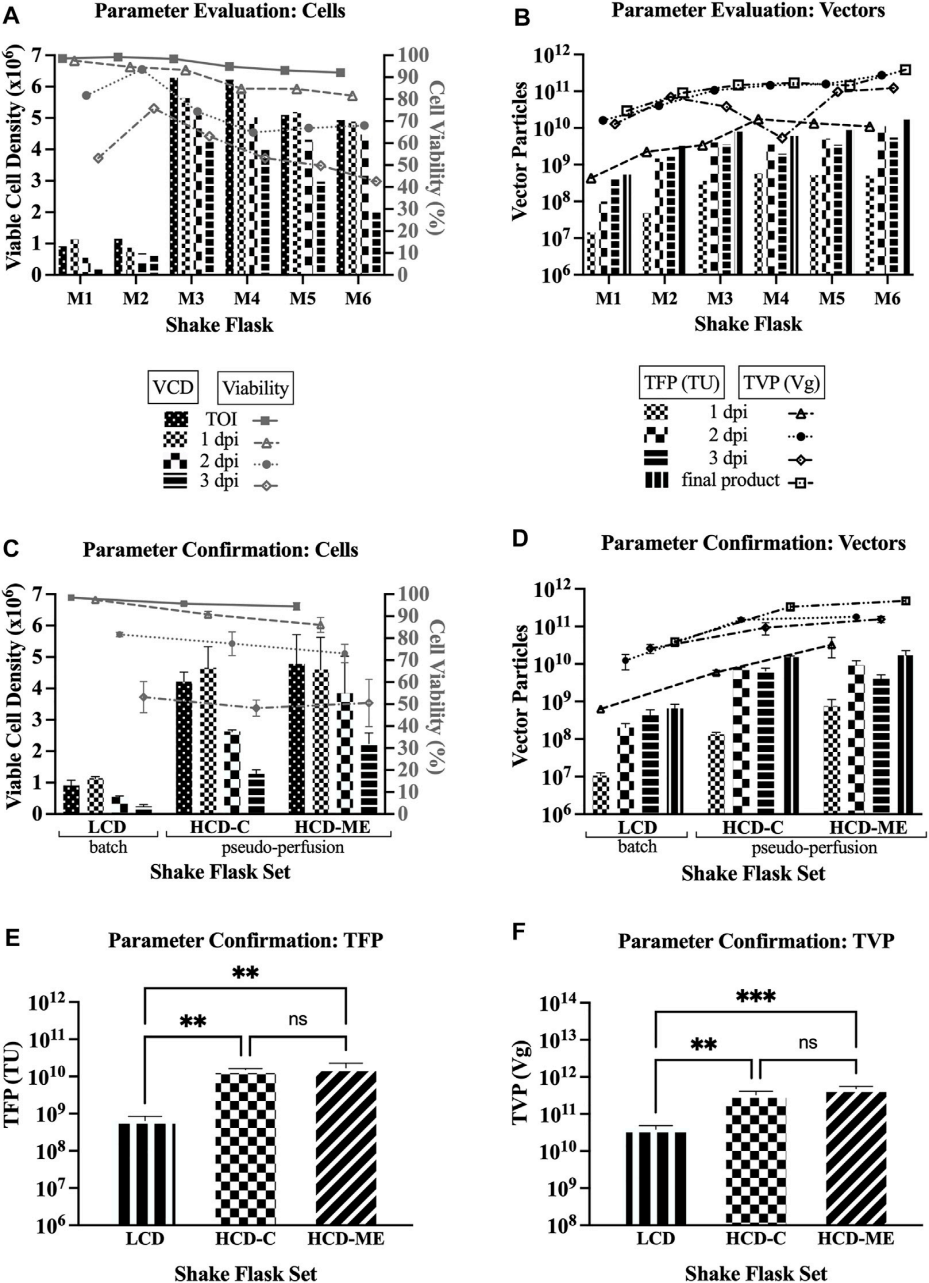
LV production at shake flask scale. VCD = viable cell density; TOI = time of induction; dpi = days post induction; LCD = low cell density at the TOI; HCD-C = high cell density at the TOI, obtained by one-step concentration; HCD-ME = high cell density at the TOI, obtained by daily medium exchange; pseudo-perfusion = daily medium exchange after induction to mimic perfusion at bioreactor scale; TFP = total functional particles; TVP = total vector particles; TU = transducing units; Vg = vector genome. 1, 2, and 3 dpi represent sample pools (e.g., 1 dpi includes LVs produced from 0 to 24 h post induction). **(A)** VCD and cell viability for parameter evaluation (6 single flasks: M1 = baseline conditions: LCD, batch, no feed; M2 = LCD, pseudo-perfusion; M3 = HCD-C, feed; M4 = HCD-ME, feed; M5 = HCD-ME, pseudo-perfusion; M6 = HCD-ME, pseudo-perfusion, feed). **(B)** TFP and TVP for parameter evaluation (flasks M1-M6). **(C)** VCD and cell viability for parameter confirmation (3 flasks per set x 3 sets: LCD baseline conditions; HCD-C + pseudo-perfusion + feed; HCD-ME + pseudo-perfusion + feed; values are shown as mean +SD). **(D)** TFP and TVP for parameter confirmation (*n* = 3 per set, values are shown as mean +SD). **(E)** Statistical analysis of TFP using one-way ANOVA–adjusted *p*-value for LCD vs. HCD-C is 0.0045, LCD vs. HCD-ME is 0.0024, and HCD-C vs. HCD-ME is 0.7772. **(F)** Statistical analysis of TVP using one-way ANOVA–adjusted *p*-value for LCD vs. HCD-C is 0.0024, LCD vs. HCD-ME is 0.0003, and HCD-C vs. HCD-ME is 0.0532.

For parameter evaluation in this first set of experiments (shake flasks M1-M6), Figure 1A shows a decreasing trend in both the viable cell density (VCD) and percent cell viability over the time course of LV production, and Figure 1B shows an increase of the TFP and TVP when the strategies of HCD at the TOI, pseudo-perfusion, and feeding are implemented. Overall, the results from the first set of experiments support the findings of improved yields when operating under the parameters of HCD at the TOI, medium exchange post-induction to implement pseudo-perfusion, and feeding post-induction (henceforth referred to as “selected parameters”).

The second set of experiments was designed to confirm these results and to compare once again the method in reaching HCD. The shake flasks were set up in triplicates for baseline parameters (referred to as LCD set) as previously defined for M1, selected parameters with one-step concentration as the method to reach HCD at the TOI (referred to as HCD-C set), and selected parameters with medium exchange pre-induction as the method to reach HCD at the TOI (referred to as HCD-ME set). Section B in Table 1 shows that, when compared to the average values of the baseline parameters (LCD set), the average values of the selected parameter sets: increase the TFP of the final product by 23- and 26-fold for HCD-C and HCD-ME, respectively; increase the TVP by 8.6- and 12-fold, respectively; and decrease the TVP/TFP by 2.7- and 2.0-fold, respectively.

For parameter confirmation in this second set of experiments, Figure 1C shows a similar decreasing trend in both the VCD and percent cell viability over the time course of LV production as seen in Figure 1A and Figure 1D confirms that implementing the tested parameters resulted in an increase of TFP and TVP. Taking a closer look, Figure 1D shows that the TFP of the final product of both selected parameter sets (average 1.52 × 10^10^ TFP for HCD-C set, average 1.71 × 10^10^ TFP for HCD-ME set) are 2 logs higher than the baseline parameter set (average 6.54 × 10^8^ TFP for LCD set). Figure 1D also shows an increasing trend in TVP, where the TVP of the final product of both selected parameter sets (average 3.33 × 10^11^ TVP for HCD-C set, average 4.81 × 10^11^ TVP for HCD-ME set) are 1 log higher than the baseline parameter set (average 3.89 × 10^10^ TVP for LCD set).

Ordinary one-way ANOVA was performed to compare the effect of the selected parameters on TFP and TVP. The statistical analysis revealed that there was a statistically significant difference in mean TFP (Figure 1E) between at least two groups (F (2, 6) = 21.33, *p* = 0.0019). Tukey’s HSD Test for multiple comparisons found that the mean value of TFP was significantly different between the LCD set and HCD-C set (*p* = 0.0045) as well as between the LCD set and the HCD-ME set (*p* = 0.0024). There was no statistically significant difference in TFP between HCD-C and HCD-ME (*p* = 0.7772), which makes sense since the only experimental difference between these two groups is the method in reaching HCD (i.e., one-step concentration for HCD-C and daily medium exchange for HCD-ME). The statistical analysis also revealed that there was a statistically significant difference in mean TVP (Figure 1F) between at least two groups (F (2, 6) = 41.97, *p* = 0.0003). Tukey’s HSD Test for multiple comparisons found that the mean value of TVP was significantly different between LCD and HCD-C (*p* = 0.0024) as well as between LCD and HCD-ME (*p* = 0.0003). There was no statistically significant difference in TVP between HCD-C and HCD-ME (*p* = 0.0532).

The results from the second set of experiments confirmed the findings of improved yields for LV production using Clone 92 producer cells when operating under the parameters of HCD at the TOI, medium exchange post-induction to implement pseudo-perfusion, and feeding post-induction. The actual TFP and TVP values for both shake flask experiments are presented in Supplementary Table S1.

### 3.2 LV Production in Perfusion Mode Using TFDF

Two LV production runs in perfusion mode with TFDF as the cell retention device at 3L bioreactor scale were implemented. Clone 92 cells were inoculated at 0.35 × 10^6^ cells/mL at 2 L working volume and the cells were grown in batch mode until 72 h after inoculation. Then, perfusion started at 0.5 VVD (vessel volume per day) for 3 days and ramped up to 0.75 and 1 VVD, respectively, for the following 2 days to support the HCD. At 176 h after inoculation, in which the VCD was 11.4 × 10^6^ cells/mL for perfusion run 1 (P1) and 12.3 × 10^6^ cells/mL for perfusion run 2 (P2), doxycycline and cumate were added for induction, and perfusion continued at 1 VVD for P1 and ramped up to 2 VVD for P2. The exchange rate for the LV production phase was ≤ 1.25 VVD for P1 and 2 VVD for P2. The inducers and basic feeding strategies of 6 g/L glucose and 3 mM glutamine were employed from the time of induction until the end of the run, which was 6 dpi for P1 and 4 dpi for P2. The total volume of media used for the LV production phase was 16.3 L for P1 and 16 L for P2. A final harvest step, where the cell culture in the bioreactor was concentrated, diafiltered with 1x phosphate buffered saline, and concentrated again, was performed for P1 using the same TFDF filter utilized for the LV production phase in perfusion mode. The final harvest step was not performed for P2.

**FIGURE 2:**
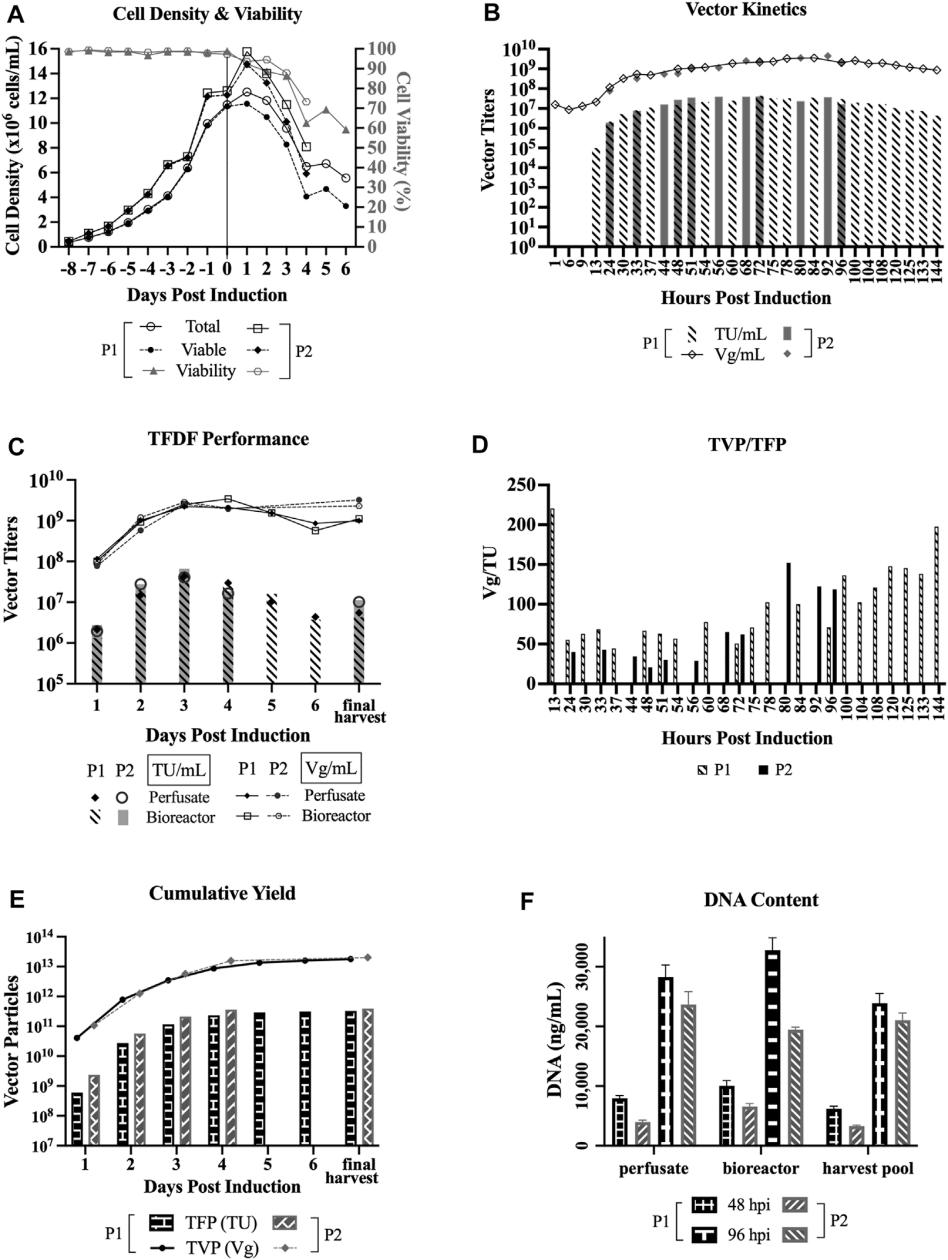
LV production in perfusion mode using TFDF. TFDF = Tangential Flow Depth Filtration; P1 = perfusion run 1; P2 = perfusion run 2; TU = transducing units; Vg = vector genome; TFP = total functional particles; TVP = total vector particles. “Final harvest” refers to the material recovered from the final harvest step (i.e., concentration, diafiltration, final concentration) in P1 and the leftoever material in the bioreactor in P2 at the end of the perfusion runs. **(A)** Cell density and cell viability data of perfusion runs (*n* = 2). **(B)** LV production kinetics in TU/mL and Vg/mL (some common and some different time points sampled between P1 and P2). **(C)** Perfusate and bioreactor samples in TU/mL and Vg/mL to assess whether the virus is retained by the TFDF device. **(D)** Ratio of TVP to TFP (some common and some different time points sampled between P1 and P2). **(E)** Cumulative vector yields for P1 (3.2 × 10^11^ TFP, 1.8 × 10^13^ TVP) over 6 days and for P2 (3.9 × 10^11^ TFP, 2.0 × 10^13^ TVP) over 4 days. **(F)** DNA content at an early time point (48hpi) and late time point (96hpi) of the perfusion runs (values are shown as mean +SD and refer to serial dilutions of the samples on the assay plate).

Figure 2A shows that the cell viability dropped steadily after induction, labeled as day 0 on the *x*-axis, whereas the total cell density and VCD increased at 1 dpi before dropping steadily. The perfusate was sampled at various time points to determine LV production kinetics, where the titers shown in Figure 2B represent a snapshot of the LV production at specific timepoints. As indicated in Figure 2B, there were some common and some different time points sampled between the two perfusion runs.

For P1, the functional titers for 1 hpi (hour post induction), 6 hpi, and 9 hpi were under the limit of detection of the GTA and the first detectable functional titer is 9.7 × 10^4^ TU/ml at 13 hpi. The peak functional titer is 4.4 × 10^7^ TU/mL at 72 hpi and the peak total titer is 3.6 × 10^9^ Vg/mL at 84 hpi. For P2, the peak functional titer is 4.1 × 10^7^ TU/mL at 72 hpi and the peak total titer is 4.5 × 10^9^ Vg/mL at 92 hpi. Looking at the kinetics of TVP/TFP, Figure 2D shows the lowest ratios between 24 hpi and 75 hpi for P1 and between 24 hpi and 72 hpi for P2.

The bioreactor vessel was also sampled at every 24-h interval to capture the snapshot of LV production at those specific timepoints to compare to the perfusate samples, with the goal of assessing whether the virus is retained by the TFDF device, as shown in Figure 2C. Overall, both functional and total vector titers are comparable between the perfusate and bioreactor samples, which indicates that the LVs were not retained by the TFDF device. Figures 2B and 2C show that the functional vector titers are typically 2 logs lower than total vector titers, which is the same trend observed in past LV experiments conducted at our laboratory as well as some materials produced at the National Research Canada (Transfiguracion et al., 2020).

Figure 2E shows the cumulative vectors produced during the two perfusion runs—3.3 × 10^11^ TFP and 1.8 × 10^13^ TVP over 6 days for P1; and 3.9 × 10^11^ TFP and 2.0 × 10^13^ TVP over 4 days for P2. Figure 2F shows a comparison of DNA content by the Picogreen assay of an early time point (48 hpi) and a late time point (96 hpi) between 3 sample types–perfusate and bioreactor samples that capture a snapshot of LV production at those time points, and harvest pool (i.e., 48 hpi includes LVs produced from 24 to 48 hpi, 96 hpi includes LVs produced from 72 to 96 hpi). Overall, the results show that DNA content is higher at the later stage of the perfusion bioreactor run.

### 3.3 Improving TFP and TVP With TFDF Perfusion Bioreactors

**FIGURE 3:**
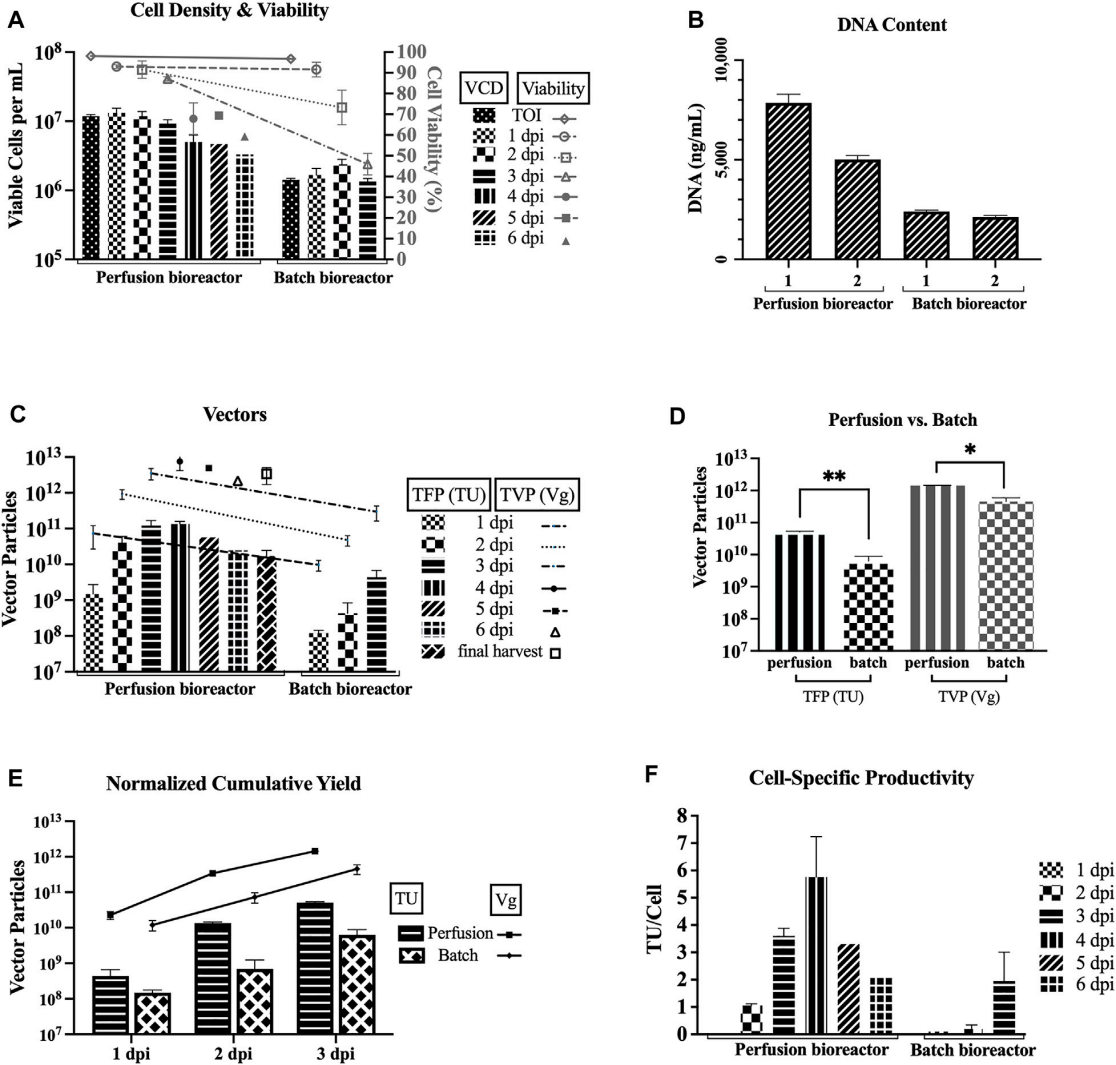
Improved TFP and TVP with TFDF perfusion bioreactors. TFDF = Tangential Flow Depth Filtration; VCD = viable cell density; TOI = time of induction; dpi = days post induction; TFP = total functional particles; TVP = total vector particles; TU = transducing units; Vg = vector genome. 1 dpi, 2 dpi, and 3 dpi represent sample pools (e.g., 2 dpi includes LVs produced from 24 to 48 h post induction). Values are shown as mean +SD (*n* = 2 for perfusion runs, *n* = 2 for bioreactor runs). **(A)** VCD and cell viability data. **(B)** DNA content for the sample pool up to 3 dpi (i.e., includes LVs produced from 0 to 72 h post induction; values shown as mean +SD refer to serial dilutions of the samples on the assay plate). **(C)** TFP and TVP data. **(D)** Statistical analysis using an unpaired *t*-test–adjusted *p*-value for TFP between perfusion vs. batch runs is 0.0037 and adjusted *p*-value for TVP between perfusion vs. batch runs is 0.0104. **(E)** Cumulative yields normalized per 1 L of harvest (at 3dpi–perfusion runs: 1.2 × 10^11^ TFP, 3.5 × 10^12^ TVP; batch runs: 4.4 × 10^9^ TFP, 3.0 × 10^11^ TVP). **(F)** Cell-specific productivity, calculated using the total cell density at each time point for each run (peak values: 7 TU/cell and 5 TU/cell for the 4 dpi pool for perfusion runs; 3 TU/cell and 1 TU/cell for the 3 dpi pool batch runs).

Figure 3 compares the two 3L bioreactors that used the TFDF as a cell retention device (HCD of average 11.8 × 10^6^ cells/mL at the TOI, perfusion mode, basic feeding of 6 g/L glucose and 3 mM glutamine, harvested at 6 dpi for run 1 and 4 dpi for run 2) and two 1L bioreactors at baseline parameters (LCD of average 1.42 × 10^6^ cells/mL at the TOI, batch mode, no feeding, both harvested at 3 dpi). Figure 3A shows that the cell viability drops slower in the perfusion runs as compared to the batch runs. Figure 3B shows higher DNA content for the two perfusion runs as compared to the two bioreactor runs for the sample pool up to 3 dpi (i.e., includes LVs produced from 0 to 72 hpi).

Figure 3C recapitulates the TFP and TVP produced during each 24-h interval and shows higher values for perfusion. Comparing the first three sample pools, the average TFP for the perfusion runs is higher than the average TFP for the batch runs by 1 log for 1 dpi (1.5 × 10^9^ vs. 1.2 × 10^8^), by 2 logs for 2 dpi (4.1 × 10^10^ vs. 4.3 × 10^8^), and by 2 logs for 3 dpi (1.2 × 10^11^ vs. 4.4 × 10^9^). As for the TVP, the average for the perfusion runs is higher than the average TVP for the batch runs by 1 log for all three sample pools (7.4 × 10^10^ vs. 9.7 × 10^9^ for 1 dpi, 9.4 × 10^11^ vs. 4.8 × 10^10^ for 2 dpi, and 3.5 × 10^12^ vs. 3.0 × 10^11^ for 3 dpi).

An unpaired *t*-test was performed to compare the TFP and TVP attained in perfusion mode and batch mode (Figure 3D). There was a significant difference in TFP between perfusion mode (M = 5.1 × 10^10^, SD = 3.0 × 10^9^) and batch mode (M = 6.3 × 10^9^, SD = 2.5 × 10^9^); t (2) = 16.3, *p* = 0.0037). Also, there was a significant difference in TVP between perfusion mode (M = 1.4 × 10^12^, SD = 2.8 × 10^10^) and batch mode (M = 4.5 × 10^11^, SD = 1.4 × 10^11^); t (2) = 9.7, *p* = 0.0104).

To facilitate a direct comparison between the perfusion and batch runs, the cumulative yields are normalized per liter of harvest. Figure 3E shows the normalized cumulative yields of average 5.1 × 10^10^ TU/L and average 1.4 × 10^12^ Vg/L for the perfusion runs and average 6.3 × 10^9^ TU/L and average 4.5 × 10^11^ Vg/L for the bioreactor runs at 3 dpi. Overall, the perfusion runs outperformed the batch runs. Figure 3F shows the cell-specific productivity calculated using the total cell density at each time point for each run. The peak specific productivity for perfusion mode is 7 TU/cell for perfusion run 1 and 5 TU/cell for perfusion run 2 for the 4 dpi pool, and the peak specific productivity for batch mode is 3 TU/cell for batch run 1 and 1 TU/cell for batch run 2 for the 3 dpi pool.

## 4 Discussion

As the field of LVs is progressing towards large-scale manufacturing to generate sufficient material for treating patients, scalability, reproducibility, and robustness are three important aspects to consider. As such, the LV production method has shifted from transfecting multiple plasmids to transfecting packaging or inducing producer cell lines. To contribute to the efforts of shifting to a more scalable, reproducible, and robust method, we chose the Clone 92 stable producer cell line for our development work. Clone 92 produces LVs with a GFP transgene, which simplifies the analytical workflow by allowing us to take advantage of the GFP as the readout signal on the flow-cytometer to assess the functional vector particles in terms of transducibility using the gene transfer assay. In addition, early upstream work has been published for these producer cells with the acoustic filter by Manceur et al. (Manceur et al., 2017), which allows us to make comparisons with the TFDF.

By testing and evaluating basic parameters at shake flask scale, we were able to select those that lead to improved yields for LV production using Clone 92 producer cells. Instead of investing time and effort in additional cell culture strategies, we focused on implementing the perfusion bioreactor runs as soon as possible. As a proof of concept for using the TFDF as a cell retention device, we successfully demonstrated LV production in two 3L bioreactors in perfusion mode using Clone 92 at the high cell density of 11.4 × 10^6^ cells/mL and 12.3 × 10^6^ cells/mL at the time of induction. For perfusion run 1, 3.3 × 10^11^ TFP and 1.8 × 10^13^ TVP was attained over 6 days, and for perfusion run 2, 3.9 × 10^11^ TFP and 2.0 × 10^13^ TVP was attained over 4 days. We implemented a longer perfusion run 1 and sampled more aggressively in the interest of monitoring the vector kinetics in addition to testing the TFDF as a cell retention device. We chose to implement a shorter perfusion run 2 since our main goal was to confirm the utilization of the TFDF as a cell retention device.

To make a direct comparison with Manceur et al.’s highest cumulative titer from a perfusion run using the acoustic filter with the same cell line (Manceur et al., 2017), we calculated the normalized cumulative functional yield per 1 L of harvest at 5dpi. Using the TFDF as the cell retention device, we achieved 1.2 × 10^11^ TU/L in perfusion run 1, which is 1.5-fold higher than Manceur et al.’s value of 8 × 10^10^ TU/L. We believe that there is potential for further improvements with the TFDF. Addressing the metabolic needs of the cells during cell growth and LV production phases should be achievable with further optimization in upstream conditions, which can directly result in an even higher increase of the cumulative functional vector yield.

The highest cell-specific productivity for our batch bioreactors is 3 TU/cell, which is slightly lower than Manceur et al.’s highest batch bioreactor value at 4.4 TU/cell, and the highest cell-specific productivity for our TFDF perfusion bioreactors is 7 TU/cell, which is lower than their highest perfusion bioreactor value at 11.5 TU/cell. We suspect that their defined upstream conditions might have an effect on the cell-specific productivity, since a small difference is observed in the batch bioreactor and that difference became much more observable in the perfusion bioreactor. However, overall, these values are in line with the 3–10 virus/cell produced for LVs and they are still far from the specific productivity that is typically attained by the wildtype HIV-1 virus, 10^3^ virus/cell (Ramirez, 2018). This points to the complexity of the heavily modified and extremely labile nature of the HIV-1-based LV system.

Of the perfusate samples that were collected at various time points of the perfusion bioreactor runs, we observed that the peak functional titer, 4.4 × 10^7^ TU/mL (run 1) and 4.1 × 10^7^ TU/mL (run 2), is at 72 hpi and the peak total titer, 3.6 × 10^9^ Vg/mL (run 1) and 4.5 × 10^9^ Vg/mL (run 2), is at 84 hpi and 92 hpi, respectively (Figure 2B). The actual peaks may shift slightly if we were to collect the samples hourly between those time points. Furthermore, the kinetics of the ratio of total vector particles to total functional particles (TVP/TFP), as shown in Figure 2D, show lowest ratios between 24 hpi and 75 hpi, which is in line with previous reports that claimed 48 hpi to 72 hpi as the typical and ideal harvest times, as longer incubations lead to a significant decrease in LV functionality (Logan et al., 2004).

DNA content is shown to be higher in the later stage of LV production for the perfusion runs (Figure 2F), this makes sense as there is higher cell death as the LV production process continues. Another observation is that there seems to be lower DNA content in perfusion run 2 as compared to perfusion run 1. This might be attributed to the fact that a higher exchange rate was implemented, essentially clearing the waste quicker, and/or that the Protoype media (HyCell TransFx-H modified SH31192) was used for the LV production phase for perfusion run 2, whereas the commercial HyCell TransFx-H medium was used perfusion run 1. In comparing the 3 dpi product, the DNA content is higher for the perfusion runs as compared to the bioreactor runs (Figure 3B), which is probably due to the fact that there is a much higher cell density in the perfusion runs. It would be a point of interest to evaluate how effectively the downstream processing steps can clear the DNA impurities along with other impurities such as host cell proteins.

Currently popular cell retention devices on the market such as the ATF (alternating tangential flow) and the acoustic filter have limitations. For example, enveloped viruses like LVs (>100 nm) stick to the ATF filter. Given the inherent fragile nature of LVs, having to find a way to remove the virus adds an additional challenge to the LV production process. The acoustic filter, on the other hand, does not promote sticking; however, it is not scalable because of heat exchange limitations. Ultimately, the TFDF performed well as a cell retention device for perfusion, as the LVs are not retained in the device (Figure 2C), and it has the scalability potential to support large-scale manufacturing of LVs.

In addition, the TFDF provides a few other advantages. For perfusion run 1, both the perfusion and final harvest operations were performed using the same filter, which provides an added benefit of a one-unit operation. No cell debris was observed in the harvest pools during the perfusion run (i.e., perfusate material), which can be an advantage as the starting material for downstream processing. Another point of observation is that the recirculation of the cell culture through the TFDF facilitates improved mixing of the cell culture, as the samples taken from the bottom of the bioreactor vessel appears to have fewer dead cells than in batch mode.

Stability is a big challenge in LV production, as LVs lose function over time and they are sensitive to environmental factors. Finding a way to produce LVs in perfusion mode at large scale would allow for the generation of more material and the usage of a cell retention device that is adapted to the fragility of the LVs would maintain a higher number of the produced functional vectors. The novelty in the work presented in this manuscript is demonstrating two successful operations of a cell retention device that can be scaled to industrial-size bioreactors. The future of this work would be an integrated continuous process, where the LVs are harvested continuously and passed onto the capture step of downstream purification, which would greatly reduce hold times, rendering less loss of LV functionality.

## Data Availability

The original contributions presented in the study are included in the article/Supplementary Material, further inquiries can be directed to the corresponding author.
