# Supplementary material for: Production of Lentiviral Vectors Using a HEK-293 Producer Cell Line and Advanced Perfusion Processing
Source: Front Bioeng Biotechnol. 2022 Jun 14;10:887716. doi: 10.3389/fbioe.2022.887716 (PMC9237754; doi:10.3389/fbioe.2022.887716)
Supplement: Supplementary file 1 [file Table1.DOCX]

| **Shake Flask** | **Parameters** | **TFP = total functional particles (in transducing units, TU)** | | | | **TVP = total vector particles  (in vector genome copies, Vg)** | | | |
| --- | --- | --- | --- | --- | --- | --- | --- | --- | --- |
|  |  | **1 dpi** | **2 dpi** | **3 dpi** | **Final product** | **1 dpi** | **2 dpi** | **3 dpi** | **Final product** |
| ***A1. Parameter evaluation:*** | | | | | | | | | |
| M1 | Baseline conditions:  LCD, batch,  no feed | 1.43 x 10^7^ | 9.97 x 10^7^ | 4.25 x 10^8^ | 5.39 x 10^8^ | 4.36 x 10^8^ | 1.62 x 10^10^ | 1.28 x 10^10^ | 2.94 x 10^10^ |
| M2 | LCD | 4.88 x 10^7^ | 1.47 x 10^9^ | 1.64 x 10^9^ | 3.31 x 10^9^ | 2.25 x 10^9^ | 4.03 x 10^10^ | 6.94 x 10^10^ | 9.02 x 10^10^ |
|  | Pseudo-perfusion |  |  |  |  |  |  |  |  |
|  |  |  |  |  |  |  |  |  |  |
| M3 | HCD-C | 3.61 x 10^8^ | 4.05 x 10^9^ | 3.58 x 10^9^ | 7.99 x 10^9^ | 3.41 x 10^9^ | 1.07 x 10^11^ | 3.84 x 10^10^ | 1.49 x 10^11^ |
|  |  |  |  |  |  |  |  |  |  |
|  | Feed |  |  |  |  |  |  |  |  |
| M4 | HCD-ME | 5.81 x 10^8^ | 3.53 x 10^9^ | 1.98 x 10^9^ | 6.09 x 10^9^ | 1.75 x 10^10^ | 1.46 x 10^11^ | 5.32 x 10^9^ | 1.69 x 10^11^ |
|  |  |  |  |  |  |  |  |  |  |
|  | Feed |  |  |  |  |  |  |  |  |
| M5 | HCD-ME | 5.21 x 10^8^ | 5.16 x 10^9^ | 3.48 x 10^9^ | 8.91 x 10^9^ | 1.34 x 10^10^ | 1.58 x 10^11^ | 9.72 x 10^10^ | 1.43 x 10^11^ |
|  | Pseudo-perfusion |  |  |  |  |  |  |  |  |
|  |  |  |  |  |  |  |  |  |  |
| M6 | HCD-ME | 5.05 x 10^8^ | 1.11 x 10^10^ | 5.52 x 10^9^ | 1.70 x 10^10^ | 1.10 x 10^10^ | 2.73 x 10^11^ | 1.23 x 10^11^ | 3.85 x 10^11^ |
|  | Pseudo-perfusion |  |  |  |  |  |  |  |  |
|  | Feed |  |  |  |  |  |  |  |  |
|  | | | | | | | | | |
| ***B. Triplicates:*** | | | | | | | | | |
| M17 | Baseline conditions:  LCD, batch,  no feed | 1.29 x 10^7^ | 1.89 x 10^8^ | 5.94 x 10^8^ | 7.95 x 10^8^ | 5.86 x 10^8^ | 1.13 x 10^10^ | 3.34 x 10^10^ | 4.52 x 10^10^ |
| M18 |  | 9.19 x 10^6^ | 1.53 x 10^8^ | 2.77 x 10^8^ | 4.39 x 10^8^ | 6.49 x 10^8^ | 7.58 x 10^9^ | 1.93 x 10^10^ | 2.75 x 10^10^ |
| M19 |  | 9.85 x 10^6^ | 2.65 x 10^8^ | 4.53 x 10^8^ | 7.28 x 10^8^ | 6.69 x 10^8^ | 1.83 x 10^10^ | 2.51 x 10^10^ | 4.41 x 10^10^ |
| *average* | | *1.06 x 10^7^* | *2.02 x 10^8^* | *4.41 x 10^8^* | *6.54 x 10^8^* | *6.35 x 10^8^* | *1.24 x 10^10^* | *2.59 x 10^10^* | *3.90 x 10^10^* |
| M20 | HCD-C  Pseudo-perfusion Feed | 1.16 x 10^8^ | 7.89 x 10^9^ | 7.67 x 10^9^ | 1.57 x 10^10^ | 5.11 x 10^9^ | 1.71 x 10^11^ | 5.68 x 10^10^ | 3.00 x 10^11^ |
| M21 |  | 1.46 x 10^8^ | 8.10 x 10^9^ | 6.49 x 10^9^ | 1.59 x 10^10^ | 6.69 x 10^9^ | 1.44 x 10^11^ | 9.76 x 10^10^ | 2.80 x 10^11^ |
| M22 |  | 1.41 x 10^8^ | 8.18 x 10^9^ | 5.28 x 10^9^ | 1.39 x 10^10^ | 6.26 x 10^9^ | 1.38 x 10^11^ | 1.23 x 10^11^ | 4.19 x 10^11^ |
| *average* | | *1.34 x 10^8^* | *8.06 x 10^9^* | *6.48 x 10^9^* | *1.52 x 10^10^* | *6.02 x 10^9^* | *1.51 x 10^11^* | *9.25 x 10^10^* | *3.33 x 10^11^* |
| M23 | HCD-ME  Pseudo-perfusion Feed | 9.47 x 10^8^ | 1.07 x 10^10^ | 4.55 x 10^9^ | 2.10 x 10^10^ | 3.06 x 10^10^ | 1.82 x 10^11^ | 1.47 x 10^11^ | 5.29 x 10^11^ |
| M24 |  | 1.00 x 10^9^ | 1.13 x 10^10^ | 4.84 x 10^9^ | 1.97 x 10^10^ | 5.19 x 10^10^ | 1.88 x 10^11^ | 1.89 x 10^11^ | 5.16 x 10^11^ |
| M25 |  | 3.28 x 10^8^ | 5.92 x 10^9^ | 2.84 x 10^9^ | 1.05 x 10^10^ | 1.57 x 10^10^ | 1.71 x 10^11^ | 1.32 x 10^11^ | 3.99 x 10^11^ |
| *average* | | *7.58 x 10^8^* | *9.29 x 10^9^* | *4.08 x 10^9^* | *1.71 x 10^10^* | *3.27 x 10^10^* | *1.80 x 10^11^* | *1.56 x 10^11^* | *4.81 x 10^11^* |

**Table S1: TFP and TVP values for LV production at shake flask scale.** TFP = total functional particles; TVP = total vector particles; TU = transducing units; Vg = vector genome; dpi = days post induction; LCD = low cell density; HCD-C = high cell density at the TOI, obtained by one-step concentration; HCD-ME = high cell density at the TOI, obtained by daily medium exchange; pseudo-perfusion = daily medium exchange after induction to mimic perfusion at bioreactor scale; feed = 6 g/L glucose and 3 mM glutamine daily. Parameter evaluation was implemented in single flasks (M1 through M6) to explore different parameters (inducing at HCD, pseudo-perfusion, and feeding) to select the best ones leading to improved yields for LV production using Clone 92 producer cells. Parameter confirmation was implemented in triplicate flasks (M17 through M25) for 3 sets (LCD baseline conditions; HCD-C + pseudo-perfusion + feed; HCD-ME + pseudo-perfusion + feed) to confirm results.
